# Detection of Runs of Homozygosity and Identification of Candidate Genes in the Whole Genome of Tunchang Pigs

**DOI:** 10.3390/ani14020201

**Published:** 2024-01-08

**Authors:** Ziyi Wang, Ziqi Zhong, Xinfeng Xie, Feifan Wang, Deyou Pan, Qishan Wang, Yuchun Pan, Qian Xiao, Zhen Tan

**Affiliations:** 1 Hainan Key Laboratory of Tropical Animal Reproduction & Breeding and Epidemic Disease Research, School of Tropical Agriculture and Forestry, Hainan University, Haikou 570228, China; wang1450887911@163.com (Z.W.);; 2 Hainan Yazhou Bay Seed Laboratory, Yongyou Industrial Park, Yazhou Bay Sci-Tech City, Sanya 572025, China; 3 Department of Animal Science, College of Animal Science, Zhejiang University, Hangzhou 310058, China

**Keywords:** Tunchang pig, runs of homozygosity, inbreeding, selection signatures

## Abstract

**Simple Summary:**

The Tunchang pig is a local pig population in Hainan Province, China, known for its delicious meat. To develop effective conservation and utilization programs, we analyzed the genetic diversity, run of homozygosity (ROH) patterns, and the degree of inbreeding in Tunchang pigs using whole genome sequencing data. Our findings show that Tunchang pigs have high genetic diversity and we identified candidate genes associated with growth and meat quality traits. These results contribute to the sustainable conservation and utilization of Tunchang pigs.

**Abstract:**

Tunchang pigs are an indigenous pig population in China known for their high tolerance to roughage, delicious meat, and fecundity. However, the number of Tunchang pigs has been declining due to the influence of commercial breeds and African swine fever, which could potentially lead to inbreeding. To assess the inbreeding level and the genetic basis of important traits in Tunchang pigs, our research investigated the patterns in “runs of homozygosity” (ROHs) using whole genome resequencing data from 32 Tunchang pigs. The study aimed to determine the length, number, coverage, and distribution model of ROHs in Tunchang pigs, as well as genomic regions with high ROH frequencies. The results of the study revealed that a total of 20,499,374 single-nucleotide polymorphisms (SNPs) and 1953 ROH fragments were recognized in 32 individuals. The ROH fragments in Tunchang pigs were predominantly short, ranging from 0.5 to 1 megabases (Mb) in length. Furthermore, the coverage of ROHs varied across chromosomes, with chromosome 3 having the highest coverage and chromosome 11 having the lowest coverage. The genetic diversity of Tunchang pigs was found to be relatively high based on the values of *H_E_* (expected heterozygosity), *H_O_* (observed heterozygosity), *pi* (nucleotide diversity), Ne (effective population size), and MAF (minor allele frequency). The average inbreeding coefficients of Tunchang pigs, as determined by three different methods (*F_HOM_*, *F_GRM_*, and *F_ROH_*), were 0.019, 0.0138, and 0.0304, respectively. These values indicate that the level of inbreeding in Tunchang pigs is currently low. Additionally, the study identified a total of 13 ROH islands on all chromosomes, which in total contained 38,913 SNPs and 120 genes. These ROH islands included genes associated with economically important traits, including meat quality (*GYS1*, *PHLPP1*, *SLC27A5*, and *CRTC1*), growth and development (*ANKS1A*, *TAF11*, *SPDEF*, *LHB*, and *PACSIN1*), and environmental adaptation (*SLC26A7*). The findings of this research offer valuable perspectives on the present status of Tunchang pig resources and offer a reference for breeding conservation plans and the efficient utilization of Tunchang pigs in the future. By understanding the inbreeding level and genetic basis of important traits in Tunchang pigs, conservation efforts can be targeted towards maintaining genetic diversity and promoting the sustainable development of this indigenous pig population.

## 1. Introduction

Tunchang pigs (Figure S1), a native pig population from Hainan Province in China, are known to have strong fecundity and can tolerate roughage [1]. Understanding the genes associated with meat quality and reproductive traits in Tunchang pigs is crucial for their breeding. Moreover, Tunchang pigs are renowned for their delicious meat, making them highly valuable. At present, the breeding herd of Tunchang pigs reaches more than 5000 heads, and there are two large-scale breeding farms in Hainan Province. To effectively protect and utilize this excellent germplasm resource, Tunchang pigs were added to the “National List of Livestock and Poultry Genetic Resources Protection” in 2014. However, Tunchang pigs have disadvantages, such as a slow growth rate, which affect economic benefits; local farmers have not given much attention to the management of Tunchang pigs, resulting in a lack of relevant records. This lack of data may hinder accurate predictions of the inbreeding level of Tunchang pigs. Inbreeding can increase the likelihood of genetic drift and the presence of harmful recessive mutations, ultimately harming the Tunchang pig population and potentially causing the loss of valuable economic traits. Therefore, surveilling the levels of inbreeding within the population is of great significance for the conservation and enhancement of genetic diversity in Tunchang pigs.

Runs of homozygosity (ROHs) are continuous homozygous DNA fragments in a diploid genome that are inherited from a mutual ancestor and can offer details about the historical background and demographic evolution of the population [2]. The formation of ROHs is largely influenced by inbreeding, making ROHs a useful indicator for measuring an individual’s inbreeding status. Studies have shown that the length of ROHs can provide insights into genome-wide inbreeding levels [3]. Longer ROH segments are typically indicative of recent parental relatedness, while shorter ROH segments suggest the presence of an ancient common ancestor in the lineage [4]. ROHs can be used to effectively estimate the level of inbreeding across the entire genome, and there is a strong positive correlation between *F_ROH_* (fraction of the genome in ROHs) and the inbreeding coefficient based on the family line. Therefore, genomic computing based on ROHs can provide a reliable estimate of inbreeding [5]. With the advancement, maturity, and wide-spread use of resequencing technology and SNP chips, as well as the continuous reduction in cost, studies on ROH analysis in livestock and poultry genomes have emerged in recent years. Researchers have increasingly utilized ROHs to unravel the genetic mechanisms underlying important traits in various animals, including cattle [4], sheep [6], and chickens [7]. For instance, Szmatola et al. detected ROHs in native cattle from Pakistan using whole genome data and identified candidate genes within ROH islands that are relevant to economic traits [8]. Gao et al. detected ROHs in eight different breeds of native chickens using sequencing data and identified genes associated with growth and development within ROH islands [9]. Cesarani et al. studied the distribution of runs of homozygosity in five European Simmental bull populations and evaluated the relationship between three production traits (milk yield and fat and protein contents) and autozygosity; the results indicated that a strong relationship between autozygosity and production traits was detected [10]. In addition, Ruan et al. analyzed the heterozygosity of 3770 American Duroc and 2096 Canadian Duroc pigs and identified genes associated with growth, metabolism, and meat quality among the identified candidate genes [11]. However, limited information is available regarding the evaluation of ROH patterns, inbreeding levels, and the distribution of ROH islands in Tunchang pigs. Therefore, the goal of our research was to detect the ROH pattern in the Tunchang pig population, observe the degree of inbreeding, and identify candidate genes specifically associated with the Tunchang pig population within ROH islands (which would suggest these candidate genes are influenced by inbreeding). This study aims to contribute to the better protection of genetic diversity in Tunchang pigs and offer precious insights for breeding programs and the effective utilization of Tunchang pigs.

## 2. Material and Methods

### 2.1. Animals and Whole Genome Resequencing Data Collection

A total of 32 Tunchang pig individuals were included in this study, of which there were 14 ear tissue samples (three-generation unrelated) collected from a native farm in Hainan province. Whole genome resequencing data from 18 other Tunchang pig individuals were obtained from a previous study [12]. Genomic DNA was extracted from each sample using a commercial tool (Tiangen Biotech Co. Ltd., Beijing, China) following the manufacturer’s protocol. The extracted DNA was then quantified using a NanoDrop 2000 spectrophotometer (Thermo Scientific, Wilmington, DE, USA) for library construction. Libraries that passed quality checks were sequenced using DNBSEQ-T7 (BGI Tech Solutions Co., Ltd., Shenzhen, China), generating 150 bp paired end reads according to the manufacturer’s protocol. The average sequencing depth was more than 15×.

### 2.2. Genotyping and Quality Controls

After obtaining the sequencing data, raw reads were filtered using SOAPnuke (v2.1.0) [13] to remove reads containing adapters, to remove low-quality 3′ end reads with base quality scores ≤20, and to remove low-quality reads with >30% bases with a quality value ≤20 or N bases. After filtering, the remaining reads from all individuals were aligned to the pig reference genome using BWA (v0.7.17) alignment software [14]. The reference genome (Sscrofa11.1) was obtained from NCBI. The resulting alignment file was sorted using SAMtools (v1.9) software [15], and the SAM file converted by BWA was further processed into a BAM file using Picard (v1.9) software [16]. Finally, PLINK v1.90 software [17] was used for SNP quality control, and the following criteria for genotypic data were used to remove SNPs: (1) minor allele frequency (MAF) < 0.05; (2) missing rate > 0.1; (3) call rate < 0.9; (4) *p*-value for Hardy–Weinberg equilibrium > 10^−6^; (5) the calling quality < 30.

### 2.3. Analyses of Genetic Diversity and Linkage Disequilibrium

Genetic diversity in the Tunchang pig population was assessed by calculating expected heterozygosity (*H_E_*), observed heterozygosity (*H_O_*), minor allele frequency (MAF), nucleotide diversity (*pi*), and effective population size (Ne). *H_E_*, *H_O_*, and MAF were computed using PLINK v1.90 [18], while *pi* was computed using VCFTOOLS (version 0.1.16) [19]. Linkage disequilibrium (LD) decay was measured by calculating the square correlation (r^2^) between pairs of SNPs using PopLDdecay [20]. The LD decline was visualized by plotting the LD distribution using the R package ggplot2. Ne was calculated using SNeP (v1.1) software [21].

### 2.4. Identification of Runs of Homozygosity

ROH segments on all autosomes were detected for each animal using PLINK v1.90 software [18]. This software utilizes a sliding window approach to detect autozygous segments. The criteria and thresholds for defining an ROH were as follows: (1) the smallest ROH length of 500 Kb; (2) the lowest number of SNPs that constituted an ROH (l) was calculated using a method similar to that proposed by Purfield et al. [3], $l=\frac{ln\frac{\alpha }{n_{s}\times n_{i}}}{ln(1-het)}$, where *α* is the percentage of false-positive ROHs (set to 0.05 in the present study), *n_s_* is the number of SNPs per individual, *n_i_* is the number of individuals, and the *het* is the proportion of heterozygosity across all SNPs. After calculation, the minimum number of SNPs that constituted an ROH was set to 53; (3) the lowest SNP density of 1 SNP per 50 Kb; (4) a sliding window size of 50 SNPs with one SNP moved at a time; and (5) the sliding window permits a maximum of 1 heterozygous SNP and up to 5 missing SNPs. We refer to some other studies [22] for ROH segments extracted from the sequence data, which were further classified according to their length into 0.5–1 Mb, 1–1.5 Mb, 1.5–2 Mb, and >2 Mb. The total number and length of individual ROHs were counted.

### 2.5. Estimation of Genomic Inbreeding Coefficient

In this study, three methods (*F_ROH_*, *F_HOM_*, and *F_GRM_*) were used to estimate the genomic inbreeding coefficients in the Tunchang pig population: (1) (*F_ROH_*) was estimated using the method proposed by McQuillan et al. [23]. The *F_ROH_* for each animal was calculated as follows:

$$F_{ROH}=\frac{\sum L_{ROH}}{L_{auto}},$$

where ∑*L_ROH_* denotes the total length of ROH fragments on an individual’s autosomes, and *Lauto* denotes the autosomal genome length covered by the analyzed SNPs. (The length in this study was 2265.77 Mb.)

*F_HOM_* was calculated using PLINK v1.9 [18] to assess the number of observed and expected autosomal homozygous genotypes in each sample. *F_HOM_* was calculated using the following formula:

$$F_{HOM}=\frac{O-E}{L-E},$$

where *O* denotes the number of homozygous genotypes observed, *E* denotes the number of homozygous genotypes accidentally expected, and *L* denotes the total number of genotype autosomal SNPs.

Finally, the genomic inbreeding coefficient (*F_GRM_*) of each individual was calculated from the genomic Relationship Matrix (GRM) using the first of the methods proposed by VanRaden [24]. The diagonal elements of the GRM were used to obtain the *F_GRM_* values for each individual using the following formula:

$$F_{GRMj}=G_{jj}-1,$$

where *F_GRMj_* represents the coefficient of inbreeding for each individual, and *Gjj* represents the diagonal element of the genomic Relationship Matrix [25].

### 2.6. Functional Annotation of Genes

Using PLINK v1.9 [18], we identified ROH islands and calculated the frequency of each SNP within an ROH. We then estimated the percentage of SNPs present in an ROH by referring to several studies [26,27] and defined the top 1% SNPs as candidate SNPs for directional selection. All adjacent SNPs positioned within the top 1% were combined to form ROH islands. To conduct enrichment analysis of these genes, we used DAVID (ver 6.7, https://david.ncifcrf.gov/ (accessed on 1 September 2023)) [28]. Only Kyoto Encyclopedia of Genes and Genomes (KEGG) pathways and gene ontology (GO) terms with *p*-values below 0.05 were included in the analysis.

## 3. Results

### 3.1. SNP Identification

After raw data quality control and filtration, we obtained 20,499,374 SNPs from the whole genomes of 32 Tunchang pigs. These SNPs were further classified functionally to understand their distribution characteristics (Figure S2). Among the SNPs, 52,293 (35.63%) were categorized as nonsynonymous and 94,486 (64.37%) were categorized as synonymous. Additionally, 10,436,315 (44.49%) SNPs were found in intergenic regions, while 153,361 (0.65%) SNPs were located in exon regions.

### 3.2. Genetic Diversity and Linkage Disequilibrium

Table 1 summarizes the genetic diversity indicators. The mean value of expected heterozygosity (*H_E_*) was 0.313, which was slightly higher than the mean value of observed heterozygosity (*H_O_*) at 0.309. The nucleotide diversity (*pi*) was 0.00327, and the minor allele frequency (MAF) was 0.229. Approximately 20.60% of the SNPs had an MAF lower than 0.10 (Figure S3). The effective population size (Ne) was 73.

For linkage disequilibrium (LD) analysis, the mean and standard deviation of the r^2^ values were 0.10 ± 0.029. Overall, the LD values of Tunchang pigs decreased with increasing distance between SNPs (Figure S4). For distances greater than 1000 Kb, the average r^2^ was approximately 0.088.

### 3.3. Distribution of ROHs

In total, we detected 1953 runs of homozygosity (ROHs) in the 32 Tunchang pigs, averaging 61.03 ROHs by each animal, and the total mean length of ROHs possessed by each animal was 22.16 Mb. Table 2 shows the number of ROH fragments of different lengths, with an average length of 0.704 Mb per fragment varying from 0.50 Mb to 2.42 Mb. The longest ROH segment was identified on chromosome 3 (contained 3233 SNPs). Short fragments (0.5–1 Mb) made up the bulk of identified ROHs, accounting for approximately 90.53% of the total detected ROH length. The proportion of ROH > 2 Mb fragments was only 0.46% of the total ROH length, and that of short ROH genomes of Tunchang pigs was 82.77%.

**Figure 1:**
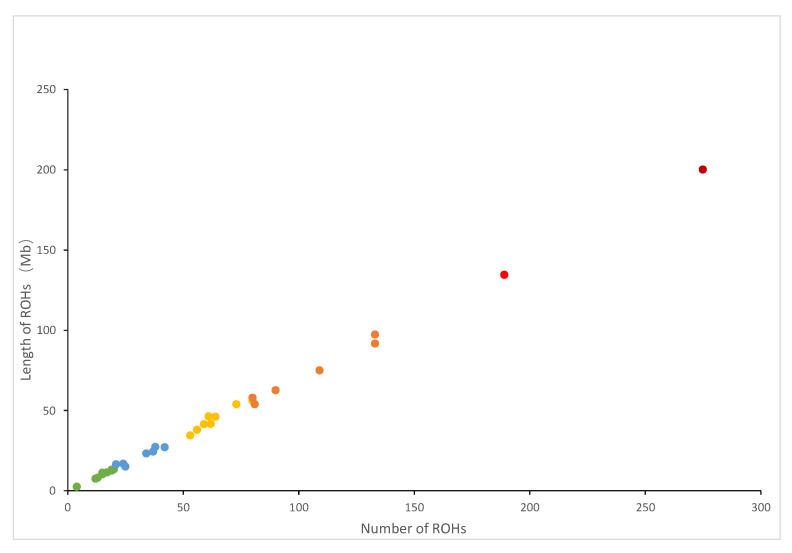
Relationship between the total number of ROH segments (*x*-axis) and the total length (Mb) of the genome in ROHs (*y*-axis) for all individuals. Each dot represents an individual.

**Figure 2:**
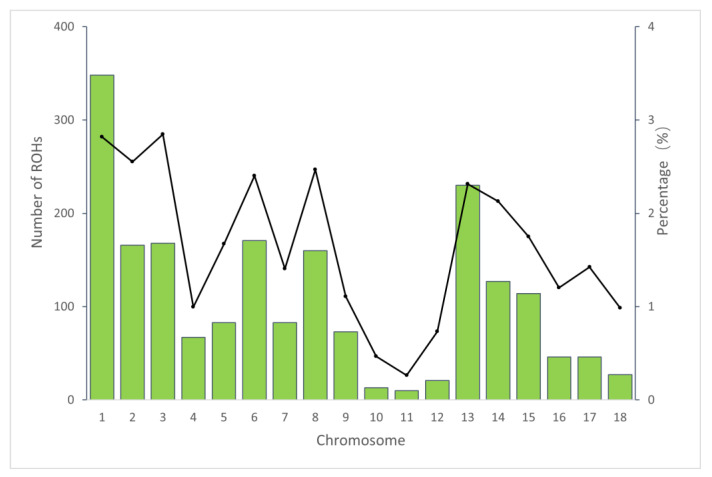
The number of ROHs and percentage coverage per chromosome in the Tunchang pig population. The vertical bars show the total number of ROHs per chromosome and the line shows the percentage of chromosomes covered with ROHs.

Figure 1 depicts the correlation between the overall number of ROHs for each individual and the overall length of genes covered by ROHs. The individual with the longest ROH had an ROH length of 200.29 Mb. Figure 2 shows the distribution of the coverage of chromosomes and the number of ROHs. Chromosome 1 had the most ROHs (348), while chromosome 11 had the fewest (10). In addition, the largest ROH coverage was observed on chromosome 3 (2.85%), while the smallest was observed on chromosome 11 (0.26%).

### 3.4. Statistics of Inbreeding Coefficients

**Figure 3:**
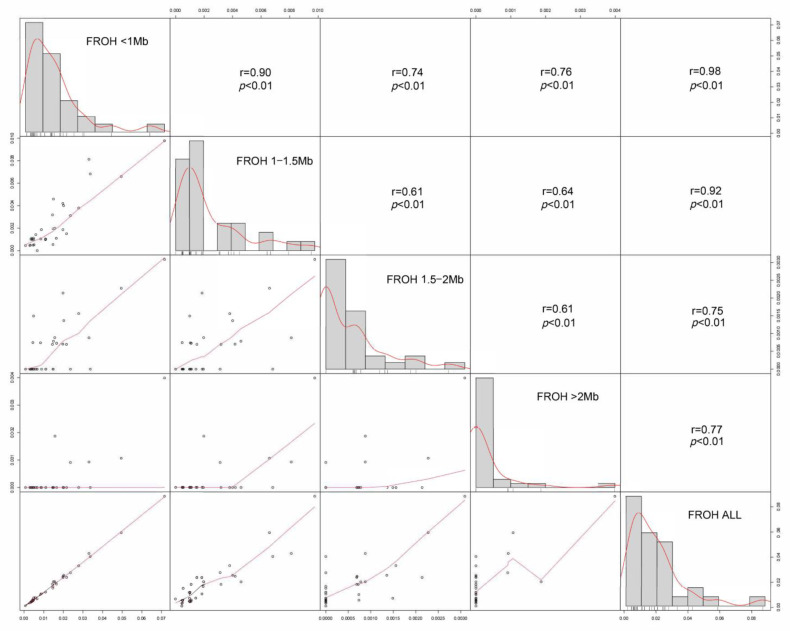
Correlation of genomic inbreeding coefficients calculated based on different-length ROH fragments (*F_ROH_* (0.5–1 Mb), *F_ROH_* (1–1.5 Mb), *F_ROH_* (1.5–2 Mb), *F_ROH_* (>2 Mb), and *F_ROH_* (ALL)). The *t*-tests of all the data show that the *p*-value between the data is less than 0.01.

Table 3 presents the results of inbreeding coefficients obtained using different calculation methods. The mean *F_ROH_* observed in the 32 individuals was 0.019, ranging from 0.001 to 0.088. The mean *F_HOM_* was 0.0138, and the mean *F_GRM_* was 0.0304. The inbreeding coefficients calculated by all three methods were relatively low, indicating a low level of inbreeding in the Tunchang pig population. Additionally, the estimated inbreeding coefficients of ROH fragments of different physical lengths varied significantly. *F_ROH_* (0.5–1 Mb) was notably higher than *F_ROH_* (1–1.5 Mb), *F_ROH_* (1.5–2 Mb), and *F_ROH_* (>2 Mb). Furthermore, a strong correlation of 0.98 was found between *F_ROH_* (0.5–1 Mb) and *F_ROH_* (All), while the weakest correlation of 0.75 was observed between *F_ROH_* (1.5–2 Mb) and *F_ROH_* (All) (Figure 3). These results suggest that short ROH fragments (0.5–1 Mb) may be the primary contributors to the calculation of *F_ROH_*.

### 3.5. ROH-Based Selective Signal Analysis

**Figure 4:**
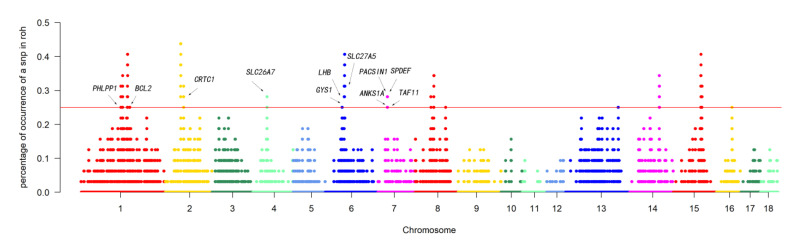
Manhattan plot of the frequency for each SNP within ROH regions among all individuals. The horizontal red line indicates the threshold for the top 1%.

We defined “ROH islands” as genomic regions with the highest frequency of ROHs. To determine the genomic regions most associated with ROHs in animals, we selected the top 1% of SNPs (range from 1 to 42,426) with the most ROHs (more than 25% of the sample) as candidate SNPs (Figure 4 and Table S1). A total of 13 ROH islands, 38,913 SNPs, and 120 genes were detected across 18 autosomes. These genomic regions ranged in length from 512.3 Kb on chromosome 1 to 2068.6 Kb on chromosome 15. Notably, the genomic region of 938.9 kb on chromosome 1 contained 106 genes.

**Figure 5:**
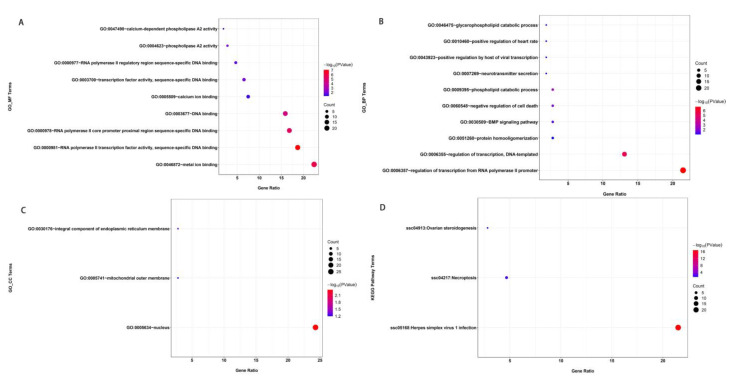
Enrichment analysis of the variants located in gene exons. The significantly enriched GO terms are classified as a molecular function (MF, (**A**)), biological process (BP, (**B**)), or cellular component (CC, (**C**)). The significantly enriched KEGG pathways are shown in (**D**). The size of each circle represents the number of genes in each GO term or pathway, and the color represents the *p*-value of each GO term or pathway.

Figure 5 illustrates the results of analyzing the functional annotations of the determined genes. A total of 22 GO terms and three pathways showed significant enrichment (Table S2). GO clustering analysis indicated that genes containing SNPs were associated with enriched catabolic processes (GO:0009395~phospholipid catabolic process and GO:0046475~glycerophospholipid catabolic process), activity processes (GO:0004623~phospholipase A2 activity), binding processes (GO:0046872~metal ion binding and GO:0005509~calcium ion binding), and DNA binding processes (GO:0003677~DNA binding; GO:0000981~RNA polymerase II transcription factor activity, sequence-specific DNA binding; GO:0003700~transcription factor activity, sequence-specific DNA binding; GO:0000978~RNA polymerase II core promoter proximal region sequence-specific DNA binding; and GO:0000977~RNA polymerase II regulatory region sequence-specific DNA binding). Furthermore, genes containing SNPs were significantly enriched in the KEGG pathways “ssc05168: Herpes simplex virus 1 infection, ssc04217: Necroptosis, and ssc04913: Ovarian steroidogenesis”.

## 4. Discussion

### 4.1. Analyses of Genetic Diversity and Linkage Disequilibrium in Tunchang Pigs

This study provides a comprehensive evaluation of genetic diversity in Tunchang pigs by assessing multiple genetic diversity parameters (heterozygosity, minor allele frequency, nucleotide diversity, and effective population size) and linkage disequilibrium (LD).

Chinese indigenous pig breeds, including Tunchang pigs, are known to exhibit higher genetic diversity compared to commercial pig breeds [26,29]. This higher genetic diversity contributes to greater heterosis in Chinese indigenous pig breeds. Consistent with expectations, the *H_O_* and *H_E_* values of Tunchang pigs (average *H_O_* = 0.31 ± 0.17, average *H_E_* = 0.31 ± 0.15) were higher than those reported for Shandong pigs (*H_O_* = 0.23), Tibetan pigs (*H_O_* = 0.23), conventional commercial pigs (*H_O_* = 0.27) [30], and Zhejiang pigs (average *H_O_* = 0.283 ± 0.185, average *H_E_* = 0.273 ± 0.162) [31]. Interestingly, the observed heterozygosity (HO) and expected heterozygosity (HE) values of Tunchang pigs were most similar to the values reported for Denmark pigs (*H_O_* = 0.31) [30] and Jiangsu pigs (*H_O_* = 0.32) [32]. The effective population size (Ne) is also a major parameter for evaluating the genetic diversity of a population, and usually a smaller value of the effective population content means less genetic variation in the population. In our study, the Ne values (73) of Tunchang pigs were higher than those reported for Liangshan pigs (15) [33] and Licha black pigs (8.7) [34]. Additionally, the mean minor allele frequency (MAF) value of Tunchang pigs was 0.23 ± 0.14, and a high proportion of SNPs had MAF values below 0.1, indicating a high level of genetic diversity [35,36]. The nucleotide diversity (*pi*) of Tunchang pigs (0.0033 ± 0.0022) was higher than that of the Bamei pig (0.0026) [37], another local Chinese pig breed. The high *pi* value suggests a rapid decay of LD in Tunchang pigs.

LD analysis provides insights into the history and evolution of a population. Comparing the level of LD between populations can reveal differences in overall genetic diversity. In our study, the distance between paired SNPs at an r^2^ value of 0.3 in Tunchang pigs was 1.7 kb, which is much smaller than that reported for commercial pig breeds, such as Changbai pigs (334 kb) and Duroc pigs (413 kb) [38], as well as other Chinese local pig breeds, such as Erhualian pigs (48.2 kb) and Jinhua pigs (170.9 kb) [39]. This suggests that the genetic diversity of Tunchang pigs is relatively stable [40]. However, continuous monitoring of genetic variation and structure is necessary to maintain diversity and prevent the influence of dynamic factors.

### 4.2. Characteristics of Runs of Homozygosity

Inbreeding can increase the homozygosity of livestock populations, but it may also lead to inbreeding depression, resulting in reduced growth rates, low reproductive capacity, and other negative effects on production performance [41]. Analyzing the distribution, length, and quantity of runs of homozygosity (ROHs) can provide insights into the inbreeding information of a population [42]. Longer ROH segments are associated with more recent inbreeding events, while shorter ROH segments indicate more distant inbreeding events [43,44]. In our study, the ROH fragments in the Tunchang pig population were predominantly short fragments (0.5–1 Mb), with a smaller proportion of longer fragments (>2 Mb), suggesting that the inbreeding level of the Tunchang pig population is relatively low; this is consistent with the results of genetic diversity, LD, and Ne analyses. However, it is important to note that ROH length is not solely dependent on inbreeding events but is also influenced by the stochastic nature of gamete formation and the rate of recombination [45].

The distribution of ROHs on chromosomes varied, with chromosome 1 having the highest number of ROHs (348). This is consistent with previous studies that have reported a positive correlation between the number of ROHs and the physical length of chromosomes [46]. The genomic regions with the highest and lowest ROH coverage in our study were on Sus scrofa chromosome 3 (SSC3) and Sus scrofa chromosome 11 (SSC11), respectively. These variations in ROH distribution may be the result of natural or artificial selection.

### 4.3. Inbreeding Coefficients

Inbreeding coefficients were estimated using different methods, including *F_ROH_*, *F_HOM_*, and *F_GRM_*. The average *F_ROH_* in the Tunchang pig population was 0.019, ranging from 0.001 to 0.088. The *F_ROH_* value for the 0.5–1 Mb ROH length category was significantly higher than the values for the other categories by *t*-test (*p*-value < 0.01). This is consistent with previous studies [47] and suggests that estimating inbreeding levels based on ROH length is a reliable method [48,49]. The results indicate that the Tunchang pig population has a relatively low level of inbreeding.

### 4.4. Candidate Genes Identified on ROH Islands

In our study, we analyzed run of homozygosity (ROH) islands in a population of Tunchang pigs and identified 13 high-frequency ROH genomic regions. The genes within these ROH hotspot regions were significantly enriched in several GO terms and KEGG pathways (*p* < 0.05). After annotation, we found that some of the GO terms were related to cellular responses. For example, the GO term “phospholipase A2 activity” has been associated with the formation of inflammatory mediators, which play important roles in inflammation, necrosis, and muscle function loss [50,51]. These GO terms may be related to the growth and development traits of Tunchang pigs. Additionally, enrichment of the KEGG pathway “Ovarian steroidogenesis” may help explain some of the reproductive traits observed in Tunchang pigs [52].

To further explore the biological functions of the candidate genes identified through ROH analysis, we conducted a literature search to uncover if any of our candidate genes have been associated with economically important traits. For example, we found the *ANKS1A* gene is known to be a negative regulator of growth factor signaling [53], the *TAF11* gene is associated with psoas muscle depth [54], and both the *SPDEF* and *PACSIN1* genes are associated with height, length, hip circumference, and weight [55,56]. We also found candidate genes related to the development and physiological processes of Tunchang pigs. For instance, the *LHB* gene plays a key role in pituitary hormone transcription and the normal development of different body parts [57]. The *BCL2* gene, as a downstream gene of the PI3K signaling pathway, may be related to the growth traits of Tunchang pigs [58]. Other genes, such as the *SLC26A7* gene involved in intracellular pH regulation, may be associated with environmental adaptation [59].

Furthermore, we detected candidate genes related to meat quality traits in Tunchang pigs. The *GYS1* gene is involved in skeletal muscle glycogen synthesis [60], the *PHLPP1* gene may be involved in bone development [61], and the *SLC27A5* gene may play a key role in intramuscular fat deposition [62]. Additionally, the *CRTC1* gene, which was enriched in our Tunchang pig population, has been reported to regulate fat deposition and indirectly affect meat quality traits [63]. These results can explain the good meat quality performance of Tunchang pigs. Overall, the regions identified in this study provide insights into the genetic theory underlying the excellent quality of Tunchang pigs.

## 5. Conclusions

In conclusion, our research provides insights into the genetic diversity, runs of homozygosity, and inbreeding coefficients in the Tunchang pig population. The results showed that the Tunchang pig population displayed a relatively high level of genetic diversity and low inbreeding-degree level. In addition, we identified candidate genes within ROH islands that may be associated with meat quality (*GYS1, PHLPP1, SLC27A5,* and *CRTC1*), growth and development (*ANKS1A, TAF11, SPDEF, LHB,* and *PACSIN1*), and environmental adaptation (*SLC26A7*). These findings contribute to our understanding of Tunchang pig resources and provide valuable information for breeding conservation and the efficient utilization of Tunchang pigs in the future.

## Figures and Tables

**Table 1:** Genetic diversity indices of Tunchang pigs.

*H_E_*	*H_O_*	MAF	*pi*	Ne
0.313 ± 0.148	0.309 ± 0.165	0.229 ± 0.141	0.00327 ± 0.00216	73

**Table 2:** Summary statistics for the number and length (in Mb) of runs of homozygosity (ROHs) categorized by ROH length classes. (ROH 0.5–1 Mb, ROH 1–1.5 Mb, ROH 1.5–2 Mb, ROH > 2 Mb, and total).

ROH Length (MB)	ROH Number	Percent (%)	Mean Length (MB)	Genome Coverage (%)
0.5–1	1768	90.53%	0.643 ± 0.121	82.77%
1–1.5	151	7.73%	1.156 ± 0.121	12.70%
1.5–2	25	1.28%	1.696 ± 0.158	3.09%
>2	9	0.46%	2.208 ± 0.259	1.45%
Total	1953	100.00%	0.704 ± 0.238	100.00%

**Table 3:** The mean genomic inbreeding coefficients (FHOM, FGRM, and FROH) for different length categories of ROHs.

*F_ROH_* (0.5–1 Mb)	*F_ROH_* (1–1.5 Mb)	*F_ROH_* (1.5–2 Mb)	*F_ROH_* (>2 Mb)	*F_ROH_* (ALL)	*F_HOM_*	*F_GRM_*
0.0157 ± 0.0147	0.0024 ± 0.0024	0.0006 ± 0.0008	0.0003 ± 0.0008	0.0190 ± 0.0181	0.0138 ± 0.1504	0.0304 ± 0.3029

## Data Availability

The raw data used in this study are publicly available and can be obtained upon reasonable request to the corresponding author.

## Supplementary Materials

The following supporting information can be downloaded at: https://www.mdpi.com/article/10.3390/ani14020201/s1, Figure S1: Figure of Tunchang Pig; Figure S2: Plotting the statistical distribution of SNP functional classes and gene regions; Figure S3: The probability density of MAF; Figure S4: The linkage disequilibrium decay of Tunchang pigs. The *x*-axis denotes SNP marker distance (kb), and the *y*-axis denotes the r^2^ between pairwise SNPs; Table S1: The specifics of the genomic regions with extended homozygosity detected in Tunchang pigs; Table S2: Significant GO terms and KEGG Pathways for genes with exonic variants.
