# Supplementary figures and images for: Detection of Runs of Homozygosity and Identification of Candidate Genes in the Whole Genome of Tunchang Pigs
Source: Animals (Basel). 2024 Jan 8;14(2):201. doi: 10.3390/ani14020201 (PMC10812771; doi:10.3390/ani14020201)

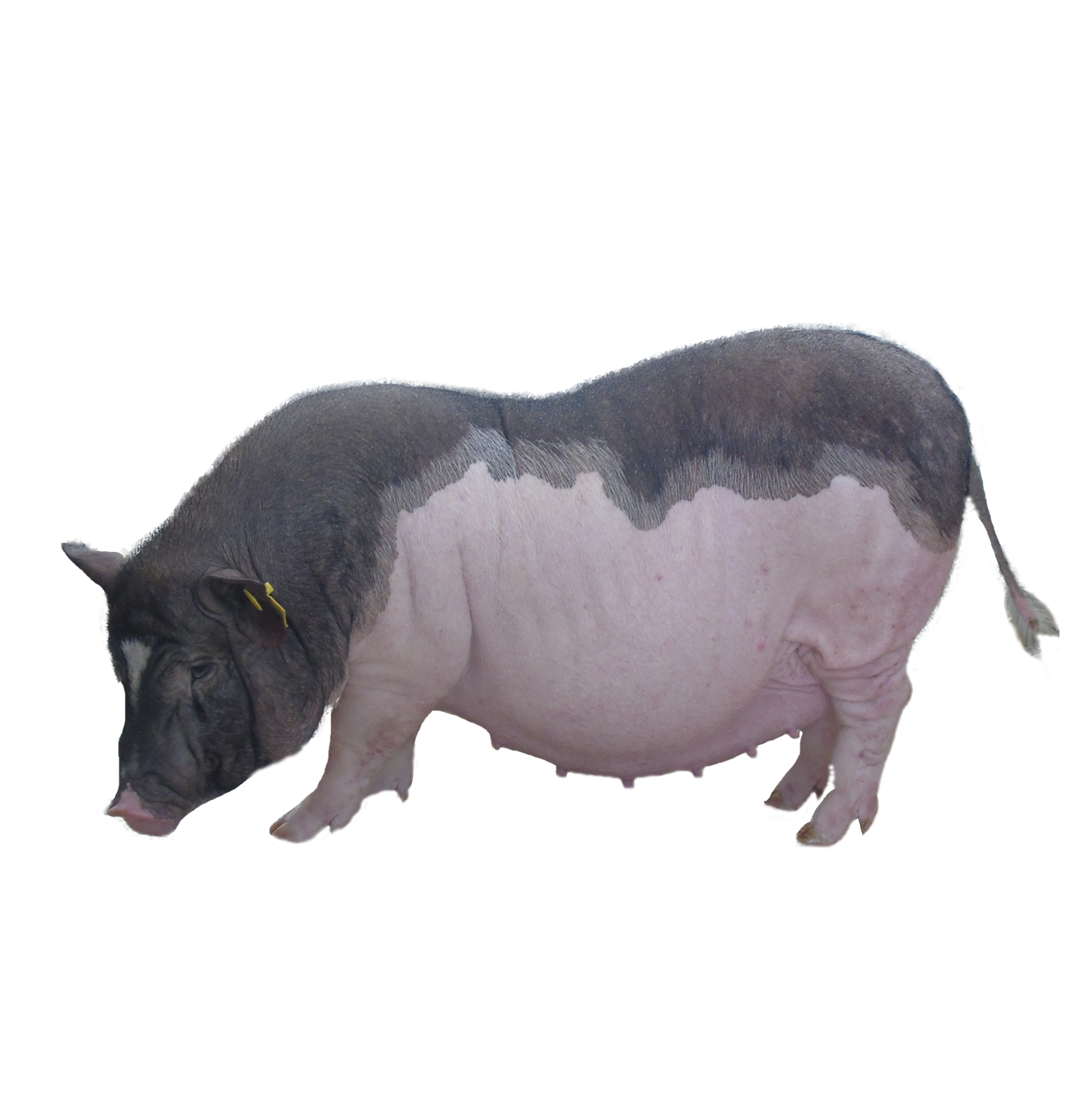

Supplement: Supplementary file 1 [file animals-14-00201-s001.zip › Figure S1.tif]

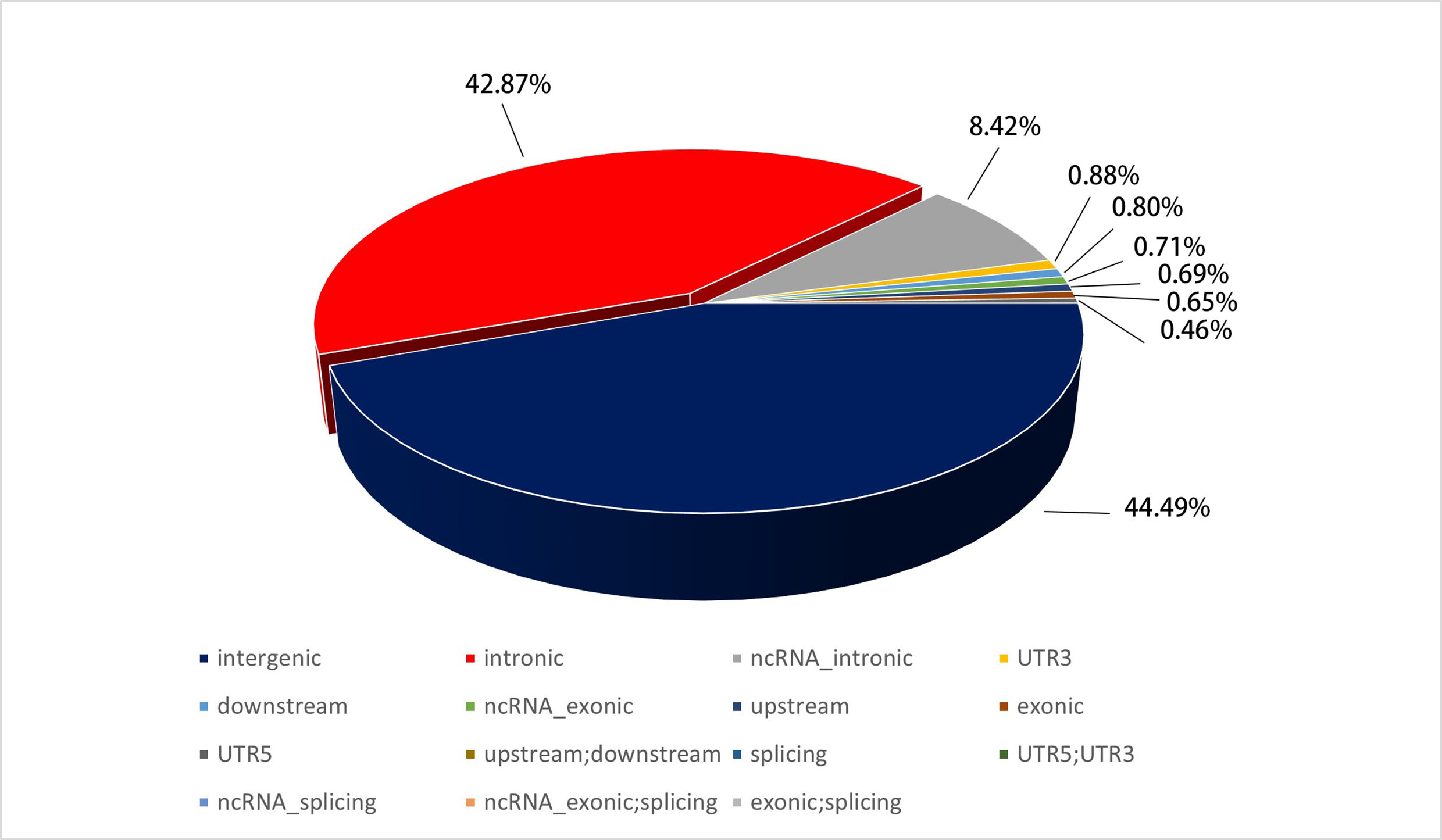

Supplement: Supplementary file 1 [file animals-14-00201-s001.zip › Figure S2 .jpg]

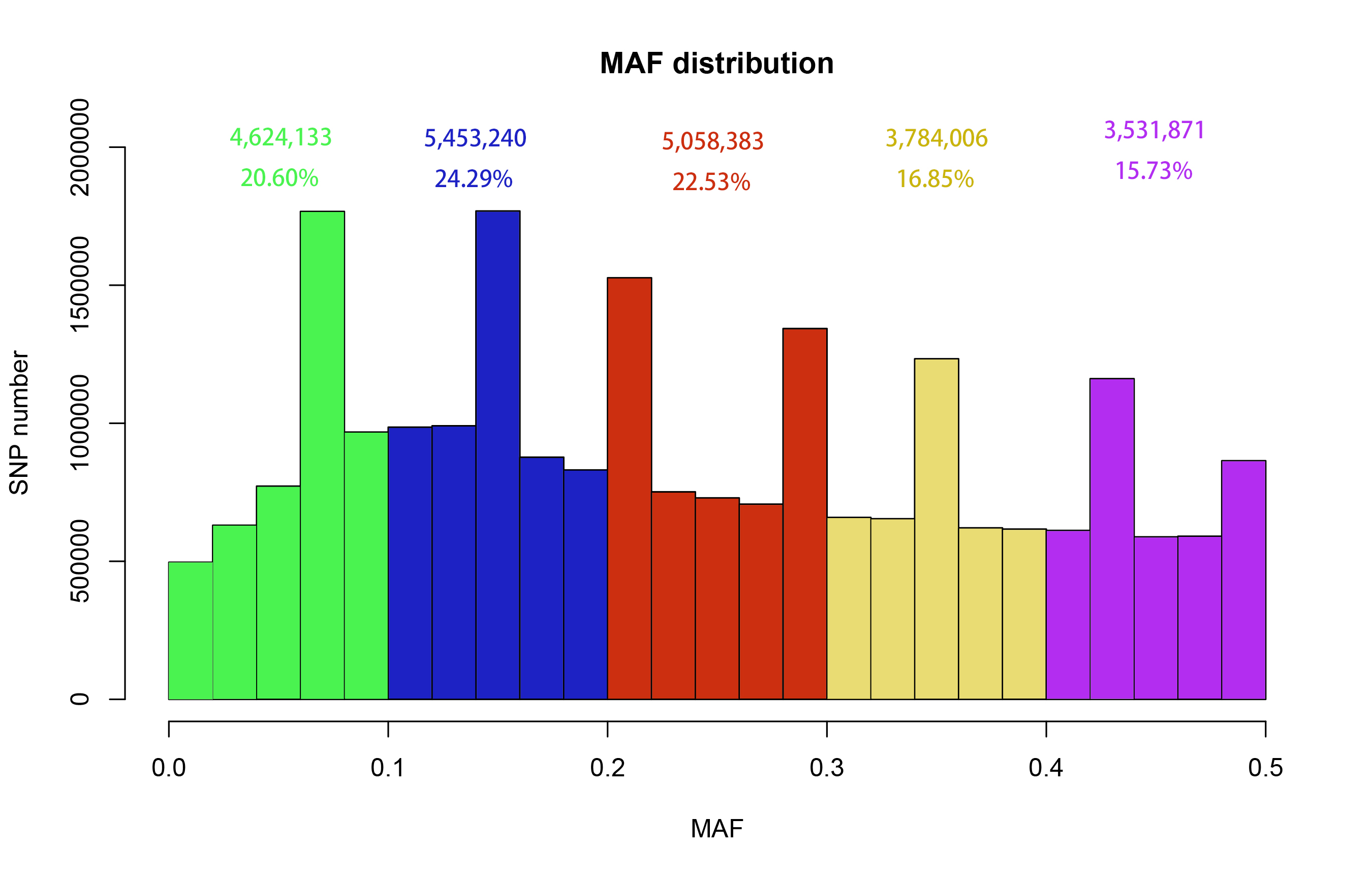

Supplement: Supplementary file 1 [file animals-14-00201-s001.zip › Figure S3.jpg]

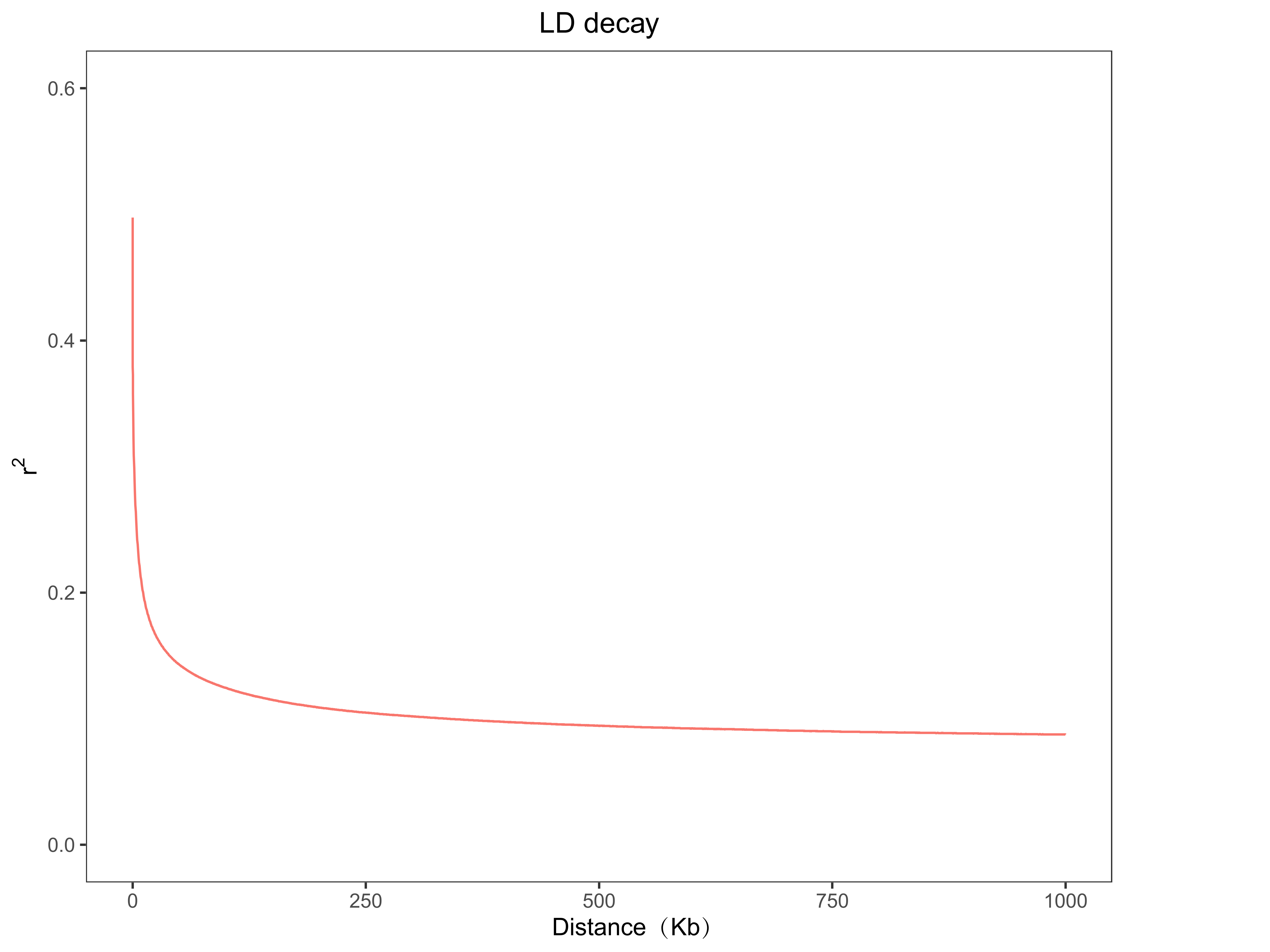

Supplement: Supplementary file 1 [file animals-14-00201-s001.zip › Figure S4.jpg]
